# Atypical Presentation of Post-Kala-Azar Dermal Leishmaniasis in Bhutan

**DOI:** 10.1155/2020/8899586

**Published:** 2020-08-21

**Authors:** Ambika Pradhan, Tashi Tobgay, Sithar Dorjee, Tenzin Wangdi, Guofa Zhou, Nadira D. Karunaweera

**Affiliations:** ^1^Jigmi Dorji Wangchuck National Referral Hospital, Thimphu, Bhutan; ^2^Khesar Gyalpo University of Medical Sciences of Bhutan, Thimphu, Bhutan; ^3^Vector-borne Disease Control Programme, Ministry of Health, Thimphu, Bhutan; ^4^Program in Public Health, University of California, Irvine, CA, USA; ^5^Department of Parasitology, Faculty of Medicine, University of Colombo, Colombo, Sri Lanka

## Abstract

This article describes an atypical case of post-kala-azar dermal leishmaniasis associated with complications due to delayed diagnosis and poor case management. The grave consequences of the prolonged disease process that continued for over 2 decades with eventual healing included facial disfigurement, visual impairment, and mental distress both to the patient and the family. The persistent infection within the skin over a lengthy period with likely increased risk of infection spread in the community highlights its potential negative impact on the ongoing leishmaniasis elimination program in the Indian subcontinent. Bhutan is a member of the leishmaniasis elimination network in Asia, and the government continues to invest in maintenance of the national healthcare system. The case study reveals the gaps in the healthcare system with hardships faced by a patient to access quality healthcare and poor patient outcome used as proxy indicators. It also points to the need to enhance access to healthcare to ensure early diagnosis and effective treatment for leishmaniasis patients including those who live in remote areas, in order to achieve the planned disease elimination targets. It also points towards the key challenges faced by a resource poor nation such as Bhutan in achieving universal health coverage and reaching the set goals for disease elimination. The findings underscore the need for a careful review of the national health care system and to address the deficiencies.

## 1. Introduction

Bhutan plans to achieve universal health coverage (UHC) early [1] and to eliminate leishmaniasis by 2020. However, leishmaniasis surveys have not been conducted since 2006 [2]. Therefore, the existing VL incidence data [3] are unreliable and are based on clinical suspicion alone. Furthermore, the actual cases may go unrecorded due to the dearth of healthcare professionals. The causative agent of VL in Bhutan is *Leishmania donovani*, closely related to the Indian subtype with several *Phlebotomus* spp. identified as probable vector(s) [4].

This article reports the first case of post-kala-azar dermal leishmaniasis (PKDL) from Bhutan with an atypical presentation. Its complex nature, prolonged history, and resultant complications underscore the need for careful review of the healthcare delivery system in Bhutan, with a focus on successful control of neglected tropical diseases (NTDs). This case adds to the evidence that, unless diagnosed early and treated effectively, leishmaniasis may result in considerable debility with devastating socioeconomic consequences and, therefore, pose a formidable challenge to achieve UHC in Bhutan.

## 2. Methodology

Patient consent was sought as per study protocol and ethics approval granted by the Research Ethic Board of Health, Bhutan. We collected information through review of medical records, laboratory reports, prescriptions maintained since 1999, and patient and family member interviews.

### 2.1. Case Description

**Figure 1:**
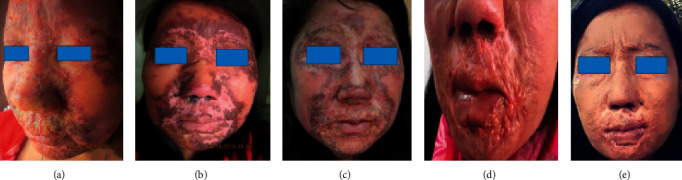
Lesion and/or residual scarring after treatment at different time points. (a) First presentation in 2014 with erythematous plaques with crusting and loss of the left eye. (b) Marked improvement after 28 doses of sodium stibogluconate (SSG) in 2014. (c) In 2017, reappearance of plaques over old healed areas. (d) In 2019, relapsed and diagnosed (and subsequently treated) as post-kala-azar dermal leishmaniasis. (e) In 2019, after treatment with miltefosine.

**Figure 2:**
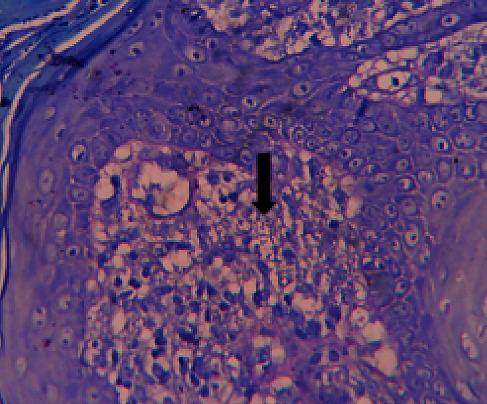
Microscopic image (×1000) of the punch biopsy tissue section stained with hematoxylin and eosin (H&E). Arrow points to Leishman-Donovan parasite bodies.

We present the case of a 37-year-old female, a mother of three children from the eastern part of Bhutan, seen by a dermatologist in 2014 at Jigme Dorji Wangchuck National Referral Hospital (JDWNRH). The patient presented with extensive erythematous plaques on forehead, central face, perioral, and cheeks that extended to both ears (Figure 1(a)). The left eye was damaged, and marked swelling was observed in the right eyelid with lid scarring and lagophthalmos. There were plaques extending to the nasal mucosa without intraoral involvement. Other skin and system examinations were apparently normal. Blood investigations showed hemoglobin of 11.7 g/dl, total leukocyte count of 6390 mm^3^, lymphocyte 28%, monocyte 4%, neutrophil 62%, eosinophils 5%, platelet count 191,000 mm^3^, ESR 39 mm/1^st^ hour, SGOT 35 units/L, SGPT 68 units/L, serum bilirubin 1 mg/dl, urea 21 mg/dl, creatinine 0.8 mg/dl, and fasting blood sugar 80 mg/dl. Tests for HIV, hepatitis B and C, and syphilis were negative. Chest radiograph, ECG, and ultrasound scan of the abdomen were normal. Slit-skin smear (SSS) stained with Giemsa showed numerous Leishman-Donovan (LD) bodies. The kala azar dip stick test (rK-39) was positive. Skin biopsy was done, which showed granulomatous inflammation with predominant infiltration of lymphocytes, a few histiocytes, and LD bodies (Figure 2).

She was treated with intramuscular injections of sodium stibogluconate (SSG) 20 mg/kg/day, given on alternate days with a total of 28 doses. Her lesions markedly improved but with residual scarring (Figure 1(b)). She again presented in 2017 with new dermal plaques over old healed lesions (Figure 1(c)). Both SSS and skin biopsy were positive for LD bodies. She responded to liposomal amphotericin B injections 2 mg/kg/day given for 28 days.

Her clinical history dated back to February 1999 when she presented at a medical clinic with fever, weight loss, and anemia. She was 4 months pregnant and had low hemoglobin (6.5 mg/dl), total leukocyte count of 3500 mm^3^, with 57% polymorphs and 43% lymphocytes, ESR 43 mm/1^st^ hour, and elevated liver enzymes. The aldehyde test was positive. Ultrasound scan of the abdomen showed hepatomegaly. Bone marrow aspirate report was unavailable. She was diagnosed as VL and treated with SSG injections 850 mg/day for 20 days. She also received 4 units of blood, and her condition improved. Seven months later, erythematous lesions appeared on her forehead that spread over the face. She was seen by a dermatologist in 2002 who recorded papules and plaques over her cheeks, periorbital, and perioral areas that were suspected as chronic dermatitis or cutaneous lupus erythematosus. She was initially treated with corticosteroids topical therapy, but subsequently, the steroids were given intralesionally and orally. In 2007, she was seen by a WHO consultant for kala azar who suspected PKDL. By this time, she had lid edema with left corneal scarring. The aldehyde test was positive, and ultrasound scan of the abdomen was normal. She was again treated with SSG 850 mg/day for 20 days. Her lesions improved only transiently. Skin biopsies in 2006, 2010, and 2013 failed to confirm leishmaniasis; therefore, no firm diagnosis was recorded. Her eye lesions progressed with the development of phthisis bulbi on the left side with swelling and scarring of the right eye lid.

She again visited the JDWNRH for follow-up in February 2019. New plaques were seen on the nostrils and chin with marked perioral scarring limiting the mouth movements (Figure 1(d)). SSS was negative for *Leishmania* parasites, and skin biopsy showed dense granulomatous inflammation in dermis with lymphocytes, histiocytes, and multinucleated giant cells with histiocytes containing small particles suggestive of LD bodies. PKDL was diagnosed, and oral miltefosine 100 mg/day was started but reduced to 50 mg/day after a month due to severe nausea and elevated liver enzymes (transaminases). Treatment continued with the reduced dosage for further 2 months with clinical improvement (Figure 1(e)) or apparent healing of lesions. No major side effects were noted.

### 2.2. Socioeconomic Aspects

The patient lives with her children and parents in a remote village of Kalapang. Her husband is a cook and lives away from home in another district. The family lives by subsistence farming. A narrow path leads to the village. Residents walk for 4-5 hours to reach the nearest town to hire a taxi to reach Mongar where the eastern regional referral hospital is located. It takes a further two-day bus ride to reach Thimphu, the capital city to access services of the dermatologist. Over a period of 20 years (1999–2019), she has visited multiple health centers incurring a considerable cost that the family could barely afford. Chronic and debilitating form of disease has had devastating impact on her and her family in terms of economic, social stigmatization, and isolation.

## 3. Discussion

PKDL that manifests as painless macular and/or papulonodular skin lesions may be a rich source of parasites, promoting transmission. Therefore, early patient management with appropriate drugs is critical to contain the infection spread [5]. Its pathophysiology is obscure and associated risk factors remain debatable [6, 7]. As per records, 10–20% in the Indian subcontinent [6, 8] and almost 50% in Sudan are affected [9] months to years after apparent drug cure of VL or as a sequel of asymptomatic infections [6]. Mucosal involvement rarely occurs [10–12]. Blepheroconjuctivitis and uveitis as sequelae are known with organisms demonstrated in extraocular/intraocular and adnexal muscles [13]. Associated eye inflammation may have grave consequences (aptly demonstrated through this case study). Diagnosis of PKDL is frequently based on clinical picture and epidemiological pattern, since the confirmation through parasite isolation has low sensitivity [14] and, nevertheless, can be improved with the use of molecular techniques [6]. Serological diagnosis (rK-39 or ELISA) is useful to detect exposure to infection, although interpretation of a positive result may be difficult due to persistence of post-VL antibodies. Poor response to antileishmanial agents and drug-resistance is widely known in PKDL with the causative agents demonstrating lower in vitro susceptibility to drugs when compared to those of VL [15]. The unacceptable treatment regimens used over the years may have led to the prolonged persistence of infection and recurrences with poor overall patient outcome described. It highlights the need for standard treatment guidelines and availability of effective treatment options in endemic areas to minimize both the resultant morbidity due to PKDL and the risk of further community spread. The latter is due to its potential to act as a reservoir for VL and trigger the emergence of infection in nonendemic areas or its reemergence in areas that have successfully eliminated the disease. PKDL is, therefore, of considerable public health significance in the region [16].

## 4. Conclusion

This case portrays the challenges faced by patients and clinicians in PKDL management, in Bhutan, which may also be applicable to other resource poor settings where the disease is generally prevalent. It highlights areas that need attention within the healthcare system of Bhutan, including the need for national level guidelines for leishmaniasis treatment and more effective disease awareness programs for public as well as healthcare personnel. Active case detection studies to assess the true burden of leishmaniasis (including PKDL) will also help to understand the magnitude of the problem. The apparent hurdles to achieve UHC in Bhutan highlight the need for remodeling of services based on primary healthcare principles to ensure equality in access to quality healthcare.
